# The role of molecular chaperones in clathrin mediated vesicular trafficking

**DOI:** 10.3389/fmolb.2015.00026

**Published:** 2015-05-19

**Authors:** Rui Sousa, Eileen M. Lafer

**Affiliations:** Department of Biochemistry and Center for Biomedical Neuroscience, University of Texas Health Science Center at San AntonioSan Antonio, TX, USA

**Keywords:** clathrin, endocytosis, chaperone, NEF, Hsp70, Hsc70, Hsp110, auxilin

## Abstract

The discovery that the 70 kD “uncoating ATPase,” which removes clathrin coats from vesicles after endocytosis, is the constitutively expressed Hsc70 chaperone was a surprise. Subsequent work, however, revealed that uncoating is an archetypal Hsp70 reaction: the cochaperone auxilin, which contains a clathrin binding domain and an Hsc70 binding J domain, recruits Hsc70^*^ATP to the coat and, concomitant with ATP hydrolysis, transfers it to a hydrophobic Hsc70-binding element found on a flexible tail at the C-terminus of the clathrin heavy chain. Release of clathrin in association with Hsc70^*^ADP follows, and the subsequent, persistent association of clathrin with Hsc70 is important to prevent aberrant clathrin polymerization. Thus, the two canonical functions of Hsp70—dissociation of existing protein complexes or aggregates, and binding to a protein to inhibit its inappropriate aggregation—are recapitulated in uncoating. Association of clathrin with Hsc70 *in vivo* is regulated by Hsp110, an Hsp70 NEF that is itself a member of the Hsp70 family. How Hsp110 activity is itself regulated to make Hsc70-free clathrin available for endocytosis is unclear, though at synapses it's possible that the influx of calcium that accompanies depolarization activates the Ca^++^/calmodulin dependent calcineurin phosphatase which then dephosphorylates and activates Hsp110 to stimulate ADP/ATP exchange and release clathrin from Hsc70^*^ADP:clathrin complexes.

## The uncoating ATPase

**Figure 1:**
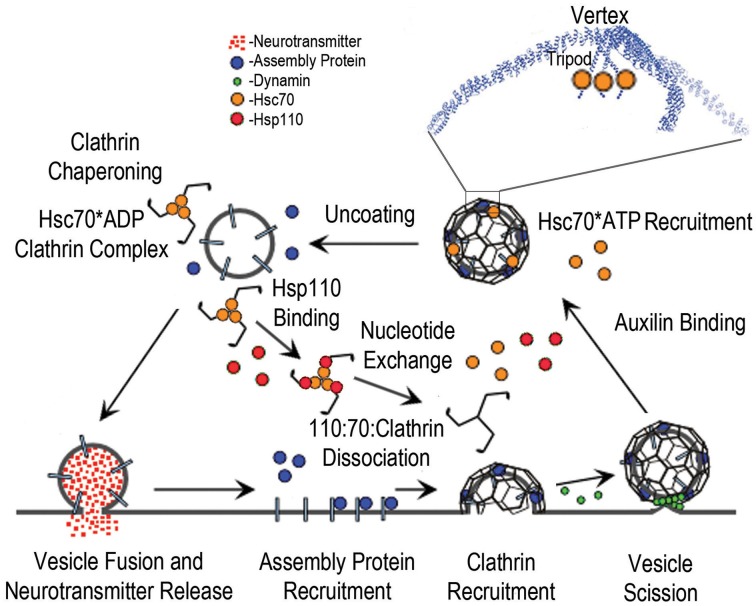
**The synaptic vesicle cycle and the roles of Hsc70, Auxilin, and Hsp110**. The cycle begins with synaptic vesicle fusion and release of neurotransmitter. Assembly/adaptor proteins then bind to the synaptic vesicle membrane proteins and phosphoinositides, and recruit clathrin; leading to membrane invagination and subsequent vesicle scission by dynamin (green spheres), followed by binding of auxilin, which recruits Hsc70^*^ATP (orange spheres) to the vesicle coat. The boxed region of the coated vesicle is expanded to show how Hsc70 binds to the flexible C-terminal tails that emerge from the helical tripod that is positioned under each vertex of the clathrin coat. Hsc70-driven disassembly (uncoating) of the coat follows, resulting in stable Hsc70^*^ADP:Clathrin complexes. Hsp110 binding then induces ADP to ATP exchange and dissociation of the Hsp110:Hsc70:Clathrin complex.

In 1984 the Rothman lab reported identification of an “uncoating ATPase” (Braell et al., 1984; Schlossman et al., 1984), an abundant 70 kD protein required to uncoat the clathrin coated vesicles that are the transient products of clathrin-mediated endocytosis at plasma membranes and clathrin-mediated intracellular traffic involving Golgi, endosomes, and lysosomes (Figure 1). The ATPase activity of the 70 kD protein was reported to be stimulated by clathrin and, by stabilizing coats with low pH or high Mg^++^ concentrations so that uncoating was blocked, it could be shown that clathrin binding by the uncoating enzyme (and stimulation of its ATPase activity) preceded the uncoating step. They showed further that the uncoating enzyme dissociated the coats into clathrin triskelia and remained associated with the triskelia after uncoating, thereby sequestering and blocking the reassembly of the triskelia into coats. Shortly thereafter, Ungewickell reported in 1985 that the uncoating ATPase appeared to be a member of the Hsp70 stress-protein family (Ungewickell, 1985).

Subsequent work by the Ungewickell group led to identification of another protein, dubbed auxilin, that associated with and promoted the assembly of clathrin coats (Ahle and Ungewickell, 1990). Auxilin was shown to be identical to a protein identified by the Eisenberg lab to be required for the Hsc70 mediated disassembly of clathrin coats (Prasad et al., 1993). Auxilin was shown to be a J cochaperone which—like DnaJ, the founding member of the J cochaperone family—contains a J domain which binds Hsc70^*^ATP (Schroder et al., 1995; Ungewickell et al., 1995; Holstein et al., 1996). The presence of both J- and clathrin-binding domains in auxilin provides the mechanism by which Hsc70 is recruited to the clathrin coat to subsequently drive its disassembly, with auxilin acting catalytically as each auxilin molecule is able to recruit multiple Hsc70 molecules to the coated vesicle (Barouch et al., 1997). The Eisenberg group showed further that uncoating followed burst kinetics: addition of limiting amounts of Hsc70 to clathrin coats resulted in an initial, rapid burst of uncoating, followed by a slow steady-state rate (Barouch et al., 1994). These kinetics were attributed to Hsc70^*^ADP becoming stably associated with the released triskelia, so that it only becomes available to carry out further rounds of uncoating upon the slow release of ADP. ADP release then allows ATP to bind and stimulate release of Hsc70 from the clathrin.

Auxilin homologs were subsequently identified in several species including yeast, *C. elegans*, and mammals (Gall et al., 2000; Umeda et al., 2000; Greener et al., 2001). Mammalian forms include the neuronal-specific auxilin 1 and the ubiquitously expressed auxilin 2 (also called cyclin G-associated protein kinase, or GAK) (Ahle and Ungewickell, 1990; Ungewickell et al., 1995; Kanaoka et al., 1997; Kimura et al., 1997; Greener et al., 2000; Umeda et al., 2000). While the amino acid sequences of auxilins are somewhat divergent between species, they all contain both clathrin binding and J domains (Gall et al., 2000; Greener et al., 2000, 2001; Pishvaee et al., 2000; Lemmon, 2001). Interestingly however, structural studies of auxilin led to the discovery that the structural diversity amongst J domains is greater than was initially anticipated: the ~100 amino acid auxilin J domain is, by comparison to the canonical ~70 residue domain exemplified by DnaJ, extended by insertion of a long but structured loop which contributes to the interface with Hsc70 (Jiang et al., 2003). The roles of J-domain structural diversity in the specificity and regulation of Hsp70 function are still poorly understood (Hennessy et al., 2005).

A series of genetic and acute perturbation studies subsequently extended these biochemical studies *in vivo* (Pishvaee et al., 2000; Greener et al., 2001; Morgan et al., 2001; Newmyer and Schmid, 2001; Lee et al., 2005, 2006; Massol et al., 2006; Yim et al., 2010) and demonstrated that, despite the initial surprise at attributing the specialized function of vesicle uncoating to the generalist Hsc70 chaperone, vesicle uncoating both *in vitro* and *in vivo* follows what we would now describe as a canonical Hsp70 mechanism (Sousa, 2014) in which a J cochaperone binds Hsp70^*^ATP and delivers it to a substrate protein concomitant with ATP hydrolysis, formation of a stable Hsp70^*^ADP:protein substrate complex and release of the J cochaperone:Hsp70 interaction. More recent studies have shown that recapitulation of this canonical mechanism extends even to the structural details of the clathrin:Hsc70 interaction. The Hsc70 binding site on the clathrin heavy chain has been mapped to a typical Hsc70 binding sequence (“QLMLT”) which is present in an extended, flexible tail that emerges from the C-termini of each of the three helices that associate in each triskelion to form a helical tripod on the inner surface of each vertex of the clathrin coat (Figure 1) (Fotin et al., 2004; Rapoport et al., 2008; Xing et al., 2010).

## Models for Hsc70 mediated clathrin coat disassembly

While the process of auxilin-mediated Hsc70 recruitment to the clathrin coat is now well understood, the mechanism by which Hsc70, once delivered to the coat, drives its disassembly is less clear and somewhat controversial. The Smith group has proposed a sequential disassembly mechanism in which auxilin first recruits an Hsc70 molecule to each of the three clathrin heavy chains that comprise each triskelion in a coat structure (Rothnie et al., 2011). Only after 3 Hsc70s have been loaded is the triskelion released from the coat assembly. In contrast, the Kirchhausen group has obtained evidence for a mechanism in which coat disassembly can be initiated with a substoichiometric number of Hsc70s, with as few as 1 Hsc70s for every ~6 clathrin heavy chains being sufficient to initiate uncoating (Bocking et al., 2011). Moreover, this number itself is variable: clathrin coats become more stable at lower pH and it was seen that, under such conditions, more Hsc70s had to bind to initiate disassembly. Conversely, coats destabilized by higher pH or mutations that disrupted clathrin:clathrin interactions required fewer Hsc70s to bind before disassembly was observed (Bocking et al., 2014). The mechanism proposed by the Kirchhausen group also differed from that of the Smith group in being non-sequential: coat disassembly was observed to begin when a certain number of Hsc70s (determined by pH and ionic conditions which modulate coat stability) had bound the coat, but Hsc70s continued to be recruited to the coat and further accelerated its disassembly even after disassembly had begun (Bocking et al., 2011). Though the Kirchhausen group developed their mechanism based on single-molecule experiments, while the Smith lab used data from ensemble experiments which can obscure mechanistic details, the latter group's conclusion are more consistent with early data (Schmid and Rothman, 1985) indicating a 1:1 stoichiometric association of Hsc70 with clathrin heavy chain during uncoating. However, it appears likely that the Kirchhausen group is correct in their conclusions that coats can be disassembled with fewer than stoichiometric numbers of Hsc70s bound. The observation of stoichiometric binding may well be a consequence of the fact that, at high Hsc70 concentrations, auxilin recruits the chaperone to the coats faster than the coats themselves disassemble (Bocking et al., 2011). As a consequence, at high Hsc70 concentrations the end state of the disassembly reaction is triskelia with Hsc70s bound 1:1 to clathrin heavy chain (3 Hsc70s per triskelion), but experiments carried out at lower Hsc70 concentrations reveal that coats can be disassembled with substoichiometric numbers of Hsc70.

Based on their structural studies of Hsc70s bound to clathrin coats, the Harrison and Kirchhausen groups proposed further that the bound Hsc70s drive disassembly by a “Brownian/Steric Wedge” mechanism. They suggested that, even in the absence of Hsc70, coats are always experiencing spontaneous fluctuations that loosen interactions between triskelia, but that such fluctuations never accumulate to a point that leads to coat disassembly (Xing et al., 2010). However, when Hsc70s are bound to the C-terminal tails under each coat vertex, they sterically block reversal of these loosening fluctuations, which then accumulate to a point where they result in coat disassembly. A different model, based on the excluded volume/entropic pulling mechanism proposed by De Los Rios and Goloubinoff (De Los Rios et al., 2006; Goloubinoff and De Los Rios, 2007) to explain how Hsp70s move proteins through channels or dissociate protein aggregates, has also been advanced. This model suggests that it is not the ability of Hsc70s to act as a passive, steric wedge that causes disassembly, but the fact that they are bound under each coat vertex by association with flexible polypeptide tethers. Such flexible tethering allows the Hsc70s to generate a disassembling force through intermolecular collisions with the walls of the coat (Lafer et al., 2014). In contrast to the “steric wedge” model, this might be described as a “wrecking ball” model for coat disassembly. Determination of which of these mechanistic models is correct awaits further experimentation.

## Control of Hsc70:clathrin chaperoning by Hsp110

The highly specialized uncoating reaction may provide insight into the mechanism of the more general proteostatic functions of the Hsp70 chaperones. Indeed, Hsc70 plays at least two roles in uncoating that may be considered analogous to its functions in supporting native protein folding. First, the disassembly of the clathrin coat may be considered analogous to reactions in which Hsp70s dissociate aggregates of misfolded proteins (Goloubinoff et al., 1999; Diamant et al., 2000; Ben-Zvi and Goloubinoff, 2001; Ben-Zvi et al., 2004; Shorter, 2011; Rampelt et al., 2012). Second, once disassembled, Hsc70 remains associated with triskelia (Schuermann et al., 2008) and inhibits their aberrant polymerization; i.e., Hsc70 not only disassembles coats, it also chaperones triskelia (Figure 1) via a mechanism that may be considered analogous to that by which Hsp70s sequester and inhibit aggregation of misfolded proteins (Mogk et al., 1999). Indeed, just as mutations in Hsp70 can accelerate accumulation of protein aggregates *in vivo* (Hesterkamp and Bukau, 1998), mutations in Hsc70 lead to aberrant clathrin polymerization and defects in endocytosis (Newmyer and Schmid, 2001).

The conclusion that Hsc70 not only disassembles clathrin coats, but also sequesters depolymerized clathrin implies that this chaperoning activity must be regulated so that clathrin can be released and made available as required for endocytosis. The most likely candidates for such regulators are the Hsp70 nucleotide exchange factors (NEFs), which control the association of Hsp70s with their protein substrates by stimulating the release of ADP from the Hsp70 (Packschies et al., 1997). This allows ATP to bind, which stimulates release of the substrate from the otherwise very stable Hsp70^*^ADP:protein substrate complex (Mccarty et al., 1995; Takeda and Mckay, 1996; Theyssen et al., 1996). *In vitro*, NEFs have been shown to release Hsc70 from triskelia and to accelerate the slow steady-state rate of uncoating that follows addition of limiting amounts of Hsc70 to reactions with clathrin coats and ATP (Schuermann et al., 2008), consistent with the Eisenberg group's conclusion that these burst kinetics reflect formation of stable Hsc70^*^ADP:clathrin complexes (Barouch et al., 1994). *In vitro*, both the Bag1 and Hsp110 NEFs could accelerate steady-state uncoating and Hsc70:clathrin dissociation, leaving it unclear which of these NEFs regulates clathrin:Hsc70 association *in vivo*. Recent experiments indicate that the relevant *in vivo* NEF is likely to be Hsp110, a protein that is itself a member of the Hsp70 family and the most abundant Hsp70 NEF in vertebrate brain (Morgan et al., 2013). Acute inhibition of Hsp110 at lamprey giant reticulospinal synapses was shown to inhibit endocytosis (specifically, synaptic vesicle recycling), presumably because inhibition of Hsp110 blocked its ability to stimulate nucleotide exchange and thereby blocked release of clathrin from Hsc70 (Morgan et al., 2013).

The question of how Hsc70:clathrin association is controlled so as to coordinate clathrin availability with endocytic activity may therefore reduce, at least in part, to the question of how Hsp110 activity is regulated. Currently, answers to this question must remain largely speculative, but one possible mechanism involves the dephosphin hypothesis (Cousin and Robinson, 2001) that has been developed to explain how the depolarization induced calcium influx that occurs at nerve terminals during synaptic transmission stimulates endocytosis. This hypothesis proposes that a coordinated wave of dephosphorylation of a number of proteins required for endocytosis (the “dephosphins” dynamin I, amphiphysins I and II, synaptojanin, epsin, eps15 and AP180) (Cousin et al., 2001) is driven by Ca^++^ activation of the calcium/calmodulin dependent protein phophatase calcineurin. Dephosphorylation of these proteins is proposed to stimulate their activity and thereby drive endocytosis.

Could such a mechanism regulate availability of Hsc70-free clathrin for endocytosis? Hsp110 is known to be phosphorylated, primarily at serine residues, both *in vivo* and *in vitro* (Ishihara et al., 2000). Evidence suggests that the relevant *in vivo* kinase(s) may be casein kinase II (CKII) or a kinase that has CKII-like specificity. *In vitro*, CKII phosphorylates mouse Hsp110 at S509, and this residue is also phosphorylated *in vivo* (Ishihara et al., 2003). Phosphorylation at S509 relieves the inhibitory effect of Hsp110 on Hsp70-mediated luciferase refolding *in vitro*, and an Hsp110 S509A mutant suppresses Hsp70-mediated luciferase refolding *in vivo*.

These observations require further comment as Hsp110 is usually observed, and is expected to, stimulate, rather than inhibit, Hsp70 functions. However, the net effect of stimulation of Hsp70 nucleotide exchange by Hsp110 is to shorten the lifetime of the Hsp70:protein substrate complex. If we consider an Hsp70-mediated reaction, such as refolding of misfolded proteins, it is easy to imagine that there will be an optimal lifetime for this complex that balances the reduction in second order processes like aggregation due to sequestration of misfolded proteins with Hsp70, and the rate of first order processes like refolding, which require that the misfolded protein be released from the Hsp70. Indeed, it is observed that when Hsp110 is titrated into Hsp70-mediated protein refolding reactions, there is an Hsp110 concentration that drives maximal refolding, and that Hsp110 concentrations in excess of this can slow refolding (Dragovic et al., 2006; Tzankov et al., 2008; Rampelt et al., 2012).

Since, in the experiments described above, phosphorylation of Hsp110 was observed to relieve inhibition of Hsp70-mediated luciferase refolding, this indicates that these experiments were carried out under conditions where Hsp110 was inducing Hsp70 nucleotide exchange and protein substrate release to a degree that inhibited protein refolding. This implies that phosphorylation of Hsp110 reduces its ability to act as an Hsp70 NEF, and suggests the following mechanism for how Ca^++^/calmodulin activation of the calcineurin phosphatase causes Hsc70 to release clathrin and make it available for endocytosis: in a resting synapse, clathrin may be mostly sequestered by Hsc70, which is in excess of clathrin (Morgan et al., 2013). Hsp110 will be phosphorylated (the brain has been shown to be exceptionally rich in the phosphorylated form of Hsp110 Ishihara et al., 2000), and its NEF activity will be correspondingly repressed. Upon depolarization, the influx of Ca^++^ into the pre-synaptic terminal will activate calmodulin and calcineurin, leading to a wave of dephosphorylation of multiple proteins, including Hsp110. Dephosphorylation of Hsp110 will stimulate its NEF activity and ultimately lead to increased release of clathrin from Hsc70, thus driving the clathrin-mediated endocytic retrieval of the synaptic vesicle proteins that follows the fusion of the synaptic vesicle with the plasma membrane and release of neurotransmitter (Figure 1).

**Figure 2:**
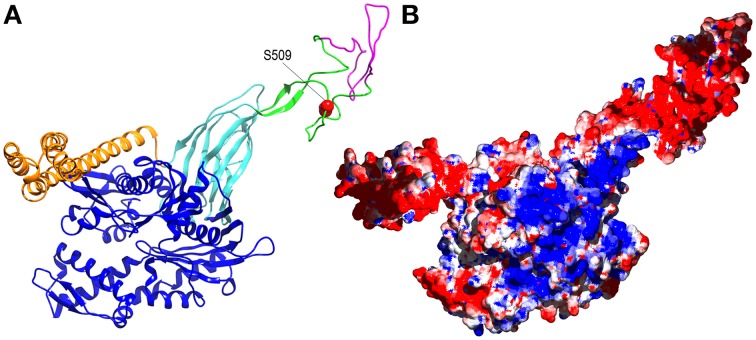
**Alternative splicing and phosphorylation of the acidic insertion loop may regulate Hsp110 activity. (A)** Ribbon model of human Hsp110 based on the crystal structure of yeast Hsp110 (pbd 3C7N Schuermann et al., 2008) with nucleotide binding domain in blue, the β-sandwich and α-helical portions of the protein binding domain in, respectively, cyan and orange, and the acidic insertion loop in green with the element that is removed by alternative splicing in magenta and the phosphorylated S509 side-chain in red. **(B)** The same model as in A but in surface representation with positively and negatively charged regions in blue and red, respectively.

How might phosphorylation regulate Hsp110 NEF activity? Again, any answer must be speculative, but it is striking that the phosphorylated S509 residue sits within the flexible ~95 residue acidic insertion loop that is positioned near the end of the β-sandwich segment of the Hsp110 protein binding domain (Figure 2A). The acidic insertion loop uniquely distinguishes the Hsp110 proteins from the more distantly related members of the Hsp70 family. There is some evidence that this loop may regulate Hsp110: the Hendershot lab observed that deletions in the insertion loop of ER Hsp110 (Grp170) affect its ability to interact with protein substrates (Pereira et al., 2014), and it has been found that the RNA encoding this loop undergoes alternative splicing (Yasuda et al., 1995). The full-length version of mouse Hsp110 (also called Hsp105α as Hsp110 has also been designated Hsp105) is apparently the predominant, constitutively expressed version of the protein, while the alternatively spliced Hsp105β, which may be expressed upon heat shock (Hatayama et al., 1994), is missing 44 residues from the loop (Figure 2A). There is no information on how this loop might regulate Hsp110 activity, but the loop's flexibility and negative charge could allow it to interact with multiple electropositive regions on the Hsp110 (or Hsp70) surface (Figure 2B), and phosphorylation might modulate these ionic interactions. All of these highly speculative mechanisms await testing by future experimentation.

## Summary

Hsc70 disassembly of clathrin coats follows a canonical Hsp70 mechanism in which a J cochaperone binds Hsp70^*^ATP and transfers it to a substrate, concomitant with ATP hydrolysis to form a stable Hsp70^*^ADP:substrate complex. After disassembling the coat, Hsc70 remains associated with clathrin to prevent its aberrant polymerization. When required for endocytosis clathrin is released from Hsc70 by a nucleotide exchange factor, whose activity may be regulated by Ca^++^ dependent dephosphorylation.

### Conflict of interest statement

The authors declare that the research was conducted in the absence of any commercial or financial relationships that could be construed as a potential conflict of interest.

## Abbreviations

Hsc70: constitutive heat-shock protein 70
Hsp70: heat-shock protein 70
NEF: nucleotide exchange factor
Hsp110: heat-shock protein 110 (aka Hsp105, Hsp105α, Hsp105β).
